# A murine model of *Trypanosoma brucei-*induced myocarditis and cardiac dysfunction

**DOI:** 10.1128/spectrum.01623-24

**Published:** 2025-01-10

**Authors:** Nathan P. Crilly, Marcelle Dina Zita, Alexander K. Beaver, Polina Sysa-Shah, Aashik Bhalodia, Kathy Gabrielson, Luigi Adamo, Monica R. Mugnier

**Affiliations:** 1 Department of Molecular and Comparative Pathobiology, Johns Hopkins University, School of Medicine 271344 , Baltimore, Maryland, USA; 2 Department of Molecular Microbiology and Immunology, Johns Hopkins Bloomberg School of Public Health 25802 , Baltimore, Maryland, USA; 3 Division of Cardiology, Department of Medicine, School of Medicine, Johns Hopkins University, Baltimore, Maryland, USA; 4 Molecular Imaging Service Center and Cancer Functional Imaging Core, School of Medicine, Johns Hopkins University, Baltimore, Maryland, USA; University of Edinburgh, Midlothian, United Kingdom

**Keywords:** cardiology, parasitology, *Trypanosoma brucei*, immunology

## Abstract

*Trypanosoma brucei* is a protozoan parasite that causes human and animal African trypanosomiases (HAT and AAT). Cardiac symptoms are commonly reported in HAT patients, and intracardiac parasites with accompanying myocarditis have been observed in both natural hosts and animal models of *T. brucei* infection. Despite the importance of *T. brucei* as a cause of cardiac dysfunction and the dramatic socioeconomic impact of African trypanosomiases in sub-Saharan Africa, there are currently no reproducible murine models of *T. brucei-*associated cardiomyopathy. We present the first clinically relevant, reproducible murine model of cardiac dysfunction in chronic *T. brucei* infection. Similar to humans, mice showed histological evidence of myocarditis and elevation of serum NT-proBNP with electrocardiographic abnormalities. Serum NT-proBNP levels were elevated prior to the development of severe ventricular dysfunction. On flow cytometry, myocarditis was associated with an increase of most myocardial immune cell populations, including multiple T cell and macrophage subsets, corroborating the notion that *T. brucei-*associated cardiac damage is an immune-mediated event. This novel mouse model represents a powerful and practical tool to investigate the pathogenesis of *T. brucei*-mediated heart damage and supports the development of therapeutic options for *T. brucei*-associated cardiac disease. In characterizing this model, we provide evidence that *T. brucei* causes cardiac disease, and we suggest that immunopathology is an important contributor to cardiac pathology. Along with other recent studies, our work demonstrates the importance of extravascular spaces, including the heart, for *T. brucei* pathogenesis.

**IMPORTANCE:**

African trypanosomiasis is a neglected tropical disease affecting both people and cattle, which represents a major public health problem in sub-Saharan Africa with an enormous socioeconomic impact. Cardiac disease represents an underappreciated clinical manifestation of African trypanosomiasis that may lead to lifelong illness despite successful treatment of infection. However, this aspect of African trypanosomiasis remains poorly understood, partially due to a lack of well-characterized and practical animal models. In this study, we present the development and characterization of a novel, reproducible, and cost-effective mouse model of cardiac dysfunction in African trypanosomiasis. We demonstrate that this model recapitulates major features of cardiac dysfunction in natural infection, including the presence of parasites in the cardiac interstitial spaces, alterations of cardiac biomarkers, and functional changes. This model represents a resource to support the understanding of cardiac complications of trypanosomiasis and the development of new therapies to prevent and treat cardiac involvement in African trypanosomiasis.

## INTRODUCTION

The threat of *Trypanosoma brucei* is a fact of life throughout sub-Saharan Africa. A protozoan parasite spread by the tsetse fly vector, *T. brucei* is the causative agent of two neglected tropical diseases, human and animal African trypanosomiasis (HAT and AAT) (1). In both humans and animals, the disease is characterized by waxing and waning fever followed by chronic wasting and a coma, which gives the disease its historical name, “sleeping sickness” (2, 3). Two subspecies are responsible for human infection: *T. b. gambiense*, which is endemic in West and Central Africa, and *T. b. rhodesiense*, which is restricted to East and Southern Africa (4). The disease character and progression are highly variable, although *T. b. rhodesiense* infection is generally aggressive and rapidly progressive, while *T. b. gambiense* infection tends to follow a more chronic, slowly progressive course (2). Although HAT is uncommon, with fewer than 1,000 cases reported in 2019 through 2020, the ubiquity of animal reservoirs and the insect vector throughout sub-Saharan Africa means that re-emergence is a constant threat (5–8). In addition to the direct impacts on human health, AAT is a severe burden to animal agriculture throughout sub-Saharan Africa and a serious contributor to food insecurity in the region, with an estimated cost of $4.75 billion per year and an incalculable impact on individual livelihoods (9).

*T. brucei* is often thought of as a parasite of the hemolymphatics and central nervous system. However, both historical and recent studies have led to a renewed appreciation for *T. brucei* as a parasite of extravascular spaces (10, 11). *T. brucei* parasites invade and colonize multiple extravascular tissues, including the heart, lungs, adipose tissue, skin, eyes, gonads, pancreas, and brain (12, 13). Within these spaces, tissue-resident parasites are associated with widespread inflammation and damage, resulting in loss of organ function that contributes to mortality (10, 11, 14).

*T. brucei* colonizes the heart, and cardiac disease is a likely contributor to HAT mortality (15, 16). HAT patients exhibit elevated cardiac NT-proBNP, a biomarker that is a sensitive predictor of heart failure, and 19% report at least one symptom consistent with heart failure (16). Although electrocardiography (EKG) is not routinely performed on HAT patients, electrocardiographic abnormalities have been reported in up to 71% of HAT patients, with most notable changes including prolonged QT interval and low voltage (16, 17). One study found that patients in Cameroon with dilated cardiomyopathy were significantly more likely to have anti-trypanosomal antibodies than healthy controls, suggesting that even clinically inapparent *T. brucei* infection might contribute to cardiac dysfunction (18). In agreement with these clinical findings, myocarditis has been reported in autopsies of patients who have died from HAT, suggesting that cardiac inflammation is a contributor to *T. brucei*-associated cardiac disease (19, 20). There is some evidence that *T. b. rhodesiense* causes more severe cardiac pathology than *T. b. gambiense*, but the small number of recorded cases makes evaluation difficult (19, 21).

Unfortunately, although there is clear evidence that cardiac disease is a consequence of African trypanosomiasis, *T. brucei*-associated heart disease remains understudied. In particular, there has been a lack of well-established animal models to allow detailed investigation into the pathology, pathogenesis, and functional consequences of *T. brucei*-associated heart disease. Several historical studies have described the histopathology of myocarditis during *T. brucei* infection, especially in large animal models such as cattle, dogs, and nonhuman primates (22–25). Although large animal models, especially primates, accurately recapitulate human disease, the expense, difficulty, and ethical issues of working with such model species have prevented their widespread adoption by *T. brucei* researchers. More recently, researchers have published a rat model of acute cardiac disease during early *T. brucei* infection. In this model, infected rats exhibited myocarditis and ventricular arrhythmias at 11 days postinfection (dpi), similar to the pathological and EKG findings reported in human patients (16, 20, 26, 27). Although the rat model demonstrates that *T. brucei*-related cardiac disease can be recapitulated in a rodent, there are important limitations. Notably, studies in the rat have been limited to the first 2 weeks of infection, while clinical data suggest that cardiac symptoms and myocarditis are most prominent during chronic HAT (19, 20). In addition, studies in the rat model have been limited to analyzing histopathology and EKG findings, although other modalities such as echocardiography and measurement of cardiac biomarkers are important for assessing the severity and progression of heart disease. Finally, there are relatively few tools for genetic and immunological manipulations in the rat, potentially limiting its usefulness for investigating disease pathogenesis (28).

A mouse model of *T. brucei*-associated cardiac disease would have multiple advantages. Compared to other animal models, mice are relatively inexpensive, safe, and simple to handle and house, making them practical for most investigators. In addition, there are numerous well-characterized strains and knockout lines of mice, allowing detailed investigation into disease mechanisms (28). Mice are also well characterized as a model species for a variety of parasitic diseases, including those caused by African (*T. brucei*) and New World (*Trypanosoma cruzi*) trypanosomes (27, 28). Historical studies performed with infection protocols that cannot be replicated in modern times showed evidence of myocarditis in *T. brucei*-infected mice via histopathology, further supporting the utility of the mouse as a model of *T. brucei*-associated cardiac disease, although the consequences of this inflammation were not evaluated (29, 30). Overall, the establishment of a robust, reproducible mouse model would allow for a detailed investigation into the mechanisms and consequences of *T. brucei*-associated cardiac disease and would be instrumental to the development of novel therapeutic options.

In this study, we present the first reproducible, clinically relevant, murine model of African trypanosomiasis optimized to characterize the functional changes and immunology of *T. brucei*-associated cardiac disease.

## RESULTS

### *T. brucei* infection induces elevation in NT-ProBNP and EKG abnormalities prior to a reduction in left ventricular ejection fraction

To evaluate the cardiac consequences of *T. brucei* infection, we infected adult male and female C57Bl/6J mice intravenously with 5–25 parasites of the *T. b. brucei* subspecies, a nonhuman infective subspecies of *T. brucei*, which is most closely related to *T. b. rhodesiense* (4). In this infection model, mice show waves of parasitemia throughout infection and systemic inflammation exemplified by significant splenomegaly (Fig. S1 and S2). Male mice exhibited a shorter survival time than female mice, although this difference was not statistically significant (Fig. S3A).

**Fig 1:**
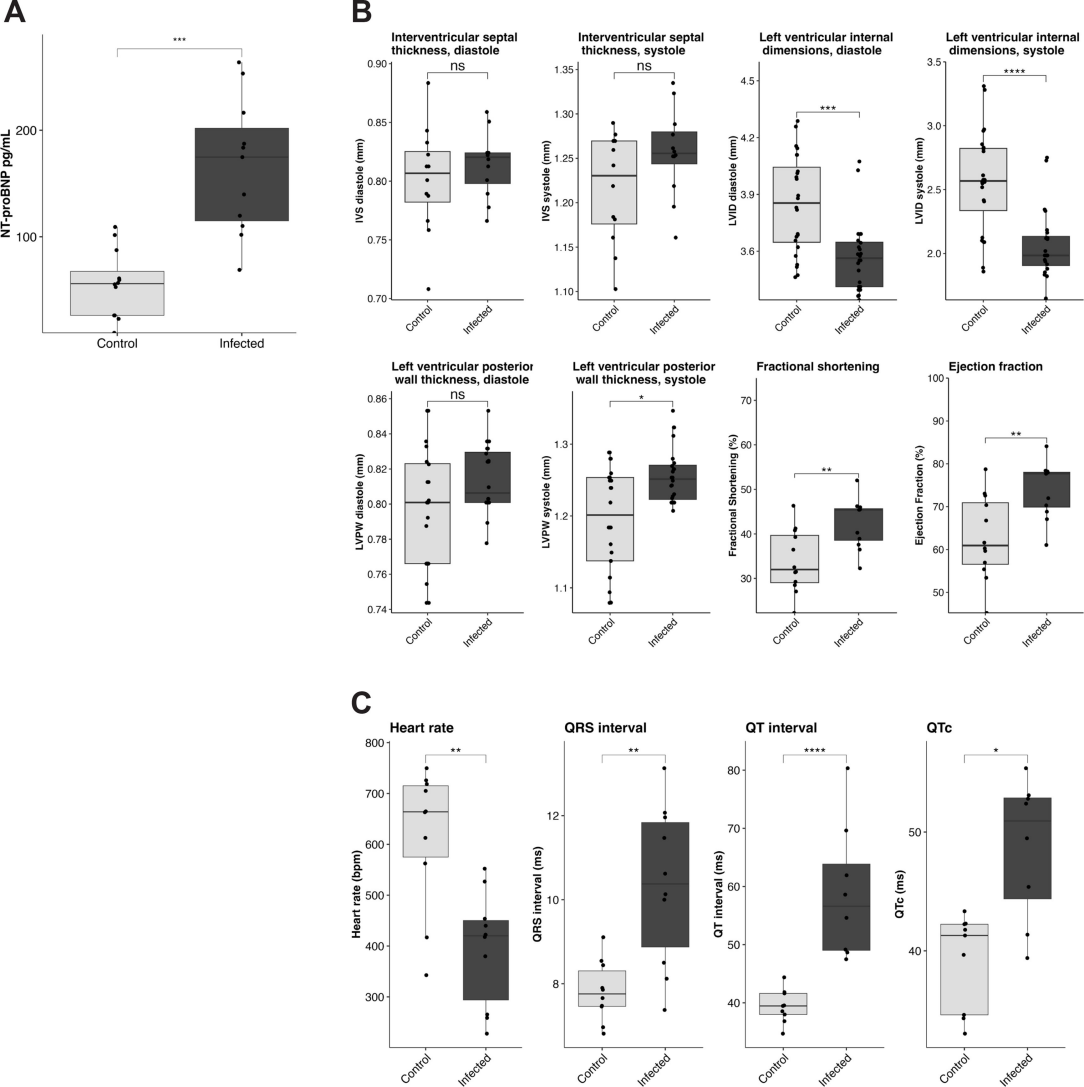
*T. brucei* infection causes elevated NT-proBNP, electrocardiographic abnormalities, and heart failure with preserved ejection fraction. (**A)** Measurement of plasma NT-proBNP at 28 dpi. Plasma NT-proBNP is significantly elevated at 28 dpi (*n* = 12) compared to age-matched uninfected controls (two-sided Student’s *t*-test, *P* = 0.00037). (**B)** Echocardiographic measurements at 28 dpi. At 28 dpi, infected mice (*n* = 12) exhibit significantly increased left ventricular ejection fraction compared to uninfected age-matched controls, as measured by sedated echocardiography (two-sided Student’s *t*-test, *P* = 0.0029). We also measured structural parameters of the left ventricle including thickness of the various left ventricular walls: interventricular septum, left ventricular posterior wall, and left ventricular anterior wall. In addition, we measured the diameter of the left ventricle at peak contraction (systole) and peak relaxation (diastole). (**C)** Electrocardiographic measurements at 28 dpi. At 28 dpi, infected mice (*n* = 10) exhibit significantly decreased heart rate (HR, two-sided Student’s *t*-test, *P* = 0.00011), increased QRS interval (two-sided Student’s *t*-test, *P* = 0.00281), increased QT interval (two-sided Student’s *t*-test, *P* = 0.00493), and increased QTc (two-sided Student’s *t*-test, *P* = 0.01294) compared to uninfected age-matched controls.

At 28 dpi, we evaluated cardiac biomarkers and function. This is a commonly used timepoint to represent chronic *T. brucei* infection (10, 14, 31–34). We measured cardiac NT-proBNP as a biomarker of cardiac dysfunction. Cardiac NT-proBNP is a sensitive marker of heart failure (35). Released from cardiomyocytes under conditions of excess stretch due to pressure or volume overload, NT-proBNP is elevated in HAT patients (16). Plasma NT-proBNP concentrations were significantly increased in infected mice at 28 dpi (Fig. 1A). NT-proBNP levels were slightly more elevated in male than in female mice, although this difference was not statistically significant (Fig. S3B).

We used echocardiography under sedation at 28 dpi to evaluate heart function. The main pumping chamber of the heart is the left ventricle. In each cardiac cycle, the left ventricle fills with blood and then contracts to eject a portion of its content. This contraction can be assessed by echocardiography and quantified as “left ventricular ejection fraction” or fractional shortening. Infected mice had both significantly increased ejection fraction and fractional shortening at 28 dpi (Fig. 1B). Ejection fraction was slightly higher in infected males than in females, although this difference was not statistically significant (Fig. S3D). We also analyzed various measurements of cardiac structure, including the thickness of the various left ventricular walls and the ventricular diameters at maximum contraction (systole) or maximum relaxation (diastole). In infected mice, we observed a decrease in the left ventricular diameter at both systole and diastole, and an increase in the thickness of the posterior wall of the left ventricle in systole (Fig. 1B). To evaluate the cardiac conduction system, we used EKGs. An EKG measures the heart’s electrical activity by electrodes applied to specific places on the skin. It is typically analyzed to assess heart rate, presence of abnormal heart rhythms (arrhythmias), and changes in the pattern of electrical conduction in the heart. The EKG records the movement (deflection) of a baseline in response to the electrical activity of the heart. Key metrics include the length of the “QRS complex” and the time between the “QRS complex” and the “T wave” (QT interval or, when corrected by heart rate, QTc). Infected mice exhibited several EKG changes. As compared to uninfected controls, infected animals had a lower heart rate. A review of the EKG tracings showed that this was due both to a reduction in the intrinsic heart rate and to a significant burden of arrhythmias, including grouped beating and dropped beats. It was also associated with significantly elevated QT intervals, including when QT interval was corrected for heart rate (QTc) (Fig. 1C). Overall, EKG findings were highly suggestive of atrioventricular (AV) node dysfunction and intermittent high-degree atrioventricular block. EKG abnormalities were generally more exaggerated in infected males than in females, although the differences were not statistically significant (Fig. S3F). All measured EKG parameters are shown in Table 1.

**TABLE 1:** Comparison of the mean values of all EKG parameters between infected animals and age-matched controls at 28 dpi (two-sided Student’s *t*-test; NS, *P* > 0.05; *, *P* ≤ 0.05; **, *P* ≤ 0.01; ***, *P* ≤ 0.001)

	Control	Infected	*P* value	Significance
RR interval (ms)	104.98	166.77	0.00111	**
Heart rate (bpm)	616.18	394.40	0.00011	***
QRS interval (ms)	7.82	10.34	0.00281	**
QT interval (ms)	39.43	58.80	0.00493	***
QTc (ms)	39.17	48.66	0.01294	*
JT interval (ms)	31.77	48.25	0.00758	**
Tpeak tend interval (ms)	28.97	42.24	0.00985	**
Q amplitude (mV)	0.01	0.02	0.42396	NS
R amplitude (mV)	0.89	0.78	0.19872	NS
S amplitude (mV)	−0.52	−0.30	0.00438	**
ST height (mV)	0.10	0.07	0.32718	NS
T amplitude (mV)	0.19	0.11	0.00670	**

**Fig 2:**
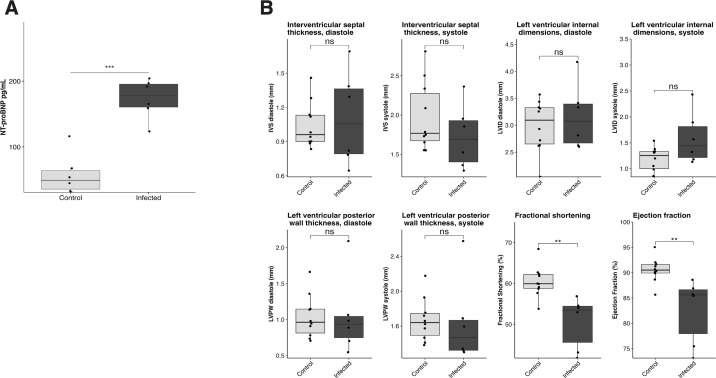
*T. brucei* infection causes elevated NT-proBNP and decreased heart function at terminal endpoint (33 dpi). (**A)** Measurement of plasma NT-proBNP at 33 dpi. Plasma NT-proBNP is significantly elevated at 33 dpi in infected mice (*n* = 6) compared to uninfected age-matched controls (two-sided Student’s *t*-test, *P* = 2.8e-05). (**B)** Echocardiographic measurements at 33 dpi. At 33 dpi, infected mice (*n* = 6) exhibit significantly decreased ejection fraction compared to uninfected age-matched controls, as measured by awake echocardiography (two-sided Student’s *t*-test, *P* = 0.011).

Plasma NT-proBNP elevation can be a marker of myocarditis. Patients with myocarditis often have a preserved ejection fraction initially but, if the disease progresses, their ejection fraction drops to a point of life-threatening cardiac dysfunction. Therefore, to further explore the cardiac consequences of *T. brucei* infection during the latest stages of infection, we repeated our functional assessments at 33 dpi, the latest timepoint at which mice can be experimentally manipulated before they begin to succumb to disease. Unsurprisingly, plasma NT-proBNP was again significantly elevated, with the mean NT-proBNP concentration (173.5 pg/mL) being similar to that observed at 28 dpi (165.4 pg/mL) (Fig. 2A). Due to the fragility of the mice at this timepoint, echocardiography was performed awake. Even so, two male mice and one female mouse had to be excluded due to reaching terminal endpoint before or during the echocardiographic evaluation. Echocardiography did not highlight any left ventricular morphological changes between infected and control mice at this timepoint (Fig. 2B). Interestingly, as hypothesized, at 33 dpi, mice exhibited a decreased ejection fraction and a decreased fractional shortening, opposite to what was seen at 28 dpi (Fig. 2B). Male mice exhibited a more marked decrease than females, although the difference was not statistically significant (Fig. S3E). This could indicate cardiac decompensation as mice approach terminal status. It should be noted that, due to the fragility of mice at 33 dpi, echocardiography was performed awake, a technique that typically results in higher measured ejection fraction. Therefore, our findings indicate a significant drop in cardiac contractility between 28 and 33 dpi.

### *T. brucei* colonizes the myocardial interstitial space and causes myocarditis

**Fig 3:**
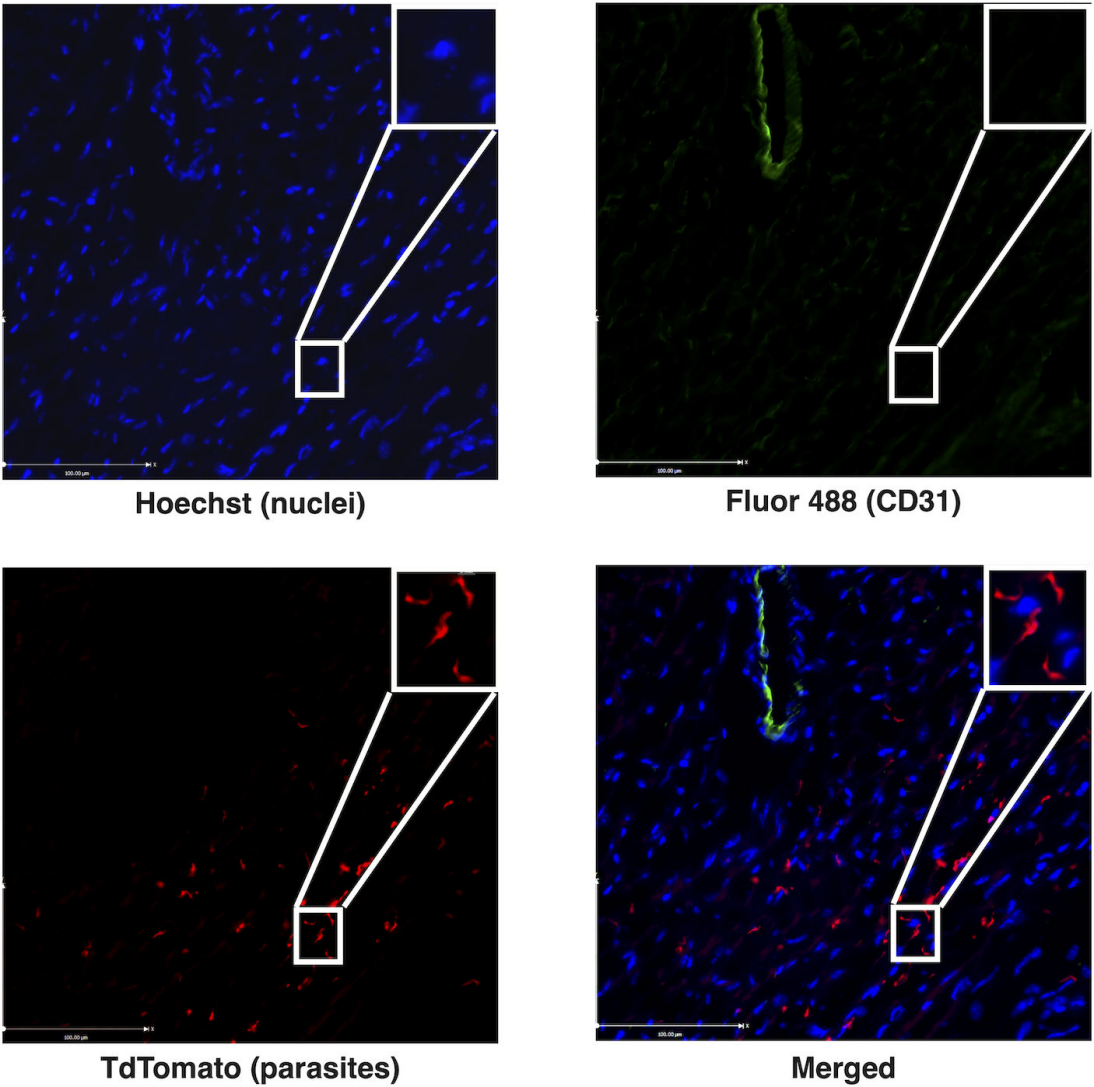
*T. brucei* parasites occupy extravascular spaces in the heart; representative immunofluorescence microphotographs of the cardiac ventricle of infected mice at 14 dpi at 20x magnification. *T. brucei* parasites (tdTomato—red) localize separately from CD31-lined vascular structures (Alexa Fluor 488—green), confirming the extravascular localization of parasites. Nuclei are stained with Hoechst (blue).

Within the mammalian host, parasites live extracellularly and colonize multiple extravascular spaces, including the heart, ultimately causing fatal disease (13, 14). To confirm the presence of intracardiac parasites in our model and to demonstrate that these parasites are extravascular, we infected adult C57Bl/6J mice with genetically modified *T. brucei* parasites that constitutively express the tdTomato fluorescent protein (36). This parasite line has previously been shown to exhibit similar infection dynamics as wild-type *T. brucei* parasites (13, 36). Mice were sacrificed at 14 dpi, a timepoint when extravascular parasite populations have been established and can be demonstrated on microscopy (10, 13). After sacrifice, we perfused mice to remove intravascular parasites, collected the heart, and performed immunofluorescence for CD31 to visualize the vasculature. *T. brucei* parasites were identified within the interstitial spaces of the heart. Intracardiac parasites localized separately from CD31-lined spaces, indicating that intracardiac parasites are extravascular (Fig. 3; Fig. S4).

**Fig 4:**
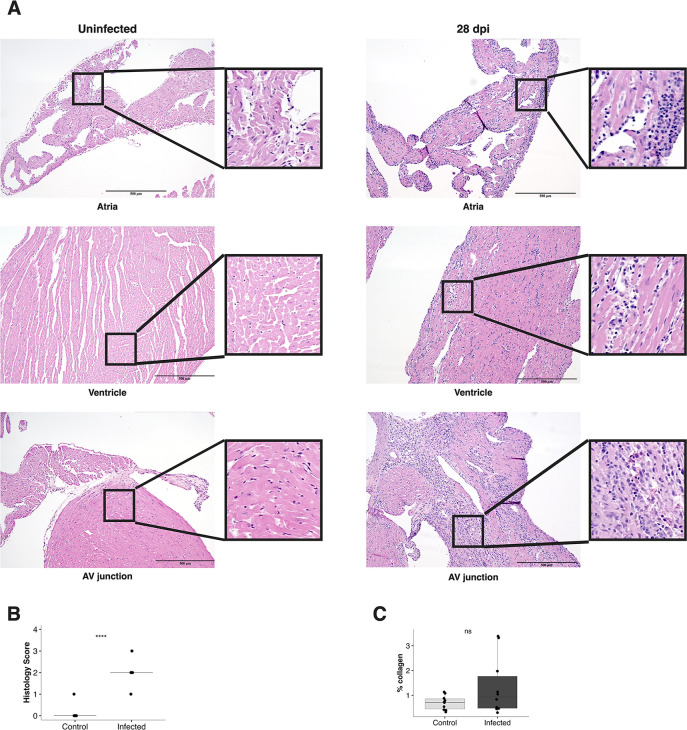
*T. brucei* infection causes myocarditis at 28 dpi. (**A)** Representative brightfield microphotographs of the heart of infected mice at 28 dpi, compared to uninfected age-matched controls at 10x magnification. Inflammation is present in all layers and regions of the heart, and is composed largely of mononuclear cells, primarily lymphocytes, plasma cells, and histiocytes. Inflammation is most severe at the atrioventricular junction. Scale bar: 500 µm. (**B)** Histological grading of myocarditis (*n* = 6) at 28 dpi, compared to equal numbers of age-matched controls. The average grade at 28 dpi is “2,” indicating multifocal moderate myocarditis without extensive associated necrosis. Histological grade is significantly higher in infected than in uninfected mice (two-sided Student’s *t*-test, *P* = 1.5e-8). (**C)** Measurement of the percentage of collagen in longitudinal sections of the heart (*n* = 6) at 28 dpi, compared to equal numbers of age-matched controls. There is no significant difference between infected and control mice (two-sided Student’s *t*-test, *P* = 0.31).

To evaluate cardiac pathology due to *T. brucei* infection, we performed histopathology on the hearts of six *T. brucei*-infected mice at 28 dpi. Of the six hearts evaluated, five met the Dallas Criteria for lymphocytic myocarditis, while the sixth exhibited borderline myocarditis (37). To further characterize histopathological changes, we used an established quantitative grading system to evaluate the severity of myocarditis (Table 2) (38). At 28 dpi, all infected mice exhibited myocarditis, with an average histological grade of 2, indicating moderate multifocal myocarditis (Fig. 4B). Inflammation affected all layers of the heart, including endocardium, myocardium, and epicardium. On histopathology, inflammatory infiltrates included large numbers of lymphocytes, plasma cells, and macrophages. Necrosis was not a major feature. The severity of inflammation was most severe in the atria and at the AV junction (Fig. 4A). The pattern and severity of inflammation observed were consistent with previous histopathology findings in humans and natural hosts (20, 22–25, 29, 39, 40). To evaluate cardiac fibrosis, longitudinal sections of the heart were stained with Masson’s Trichrome, and the percentage of collagen in the myocardium was quantified using the Automated Fibrosis Analysis Tool (AFAT) (41). At 28 dpi, infected mice had a higher percentage of collagen in the heart compared to uninfected mice, although this difference was not statistically significant (Fig. 4C).

**TABLE 2:** Histological grading criteria

Grade	Description
0	No inflammatory infiltrates
1	Limited focal distribution of myocardial lesions
2	Multiple lesions with inflammatory infiltrates and some confluence
3	Multiple lesions with confluence and extensive necrosis
4	Lesions affecting most of the observed tissue

### Immune cell populations in the heart are consistent with immune-mediated myocarditis

**Fig 5:**
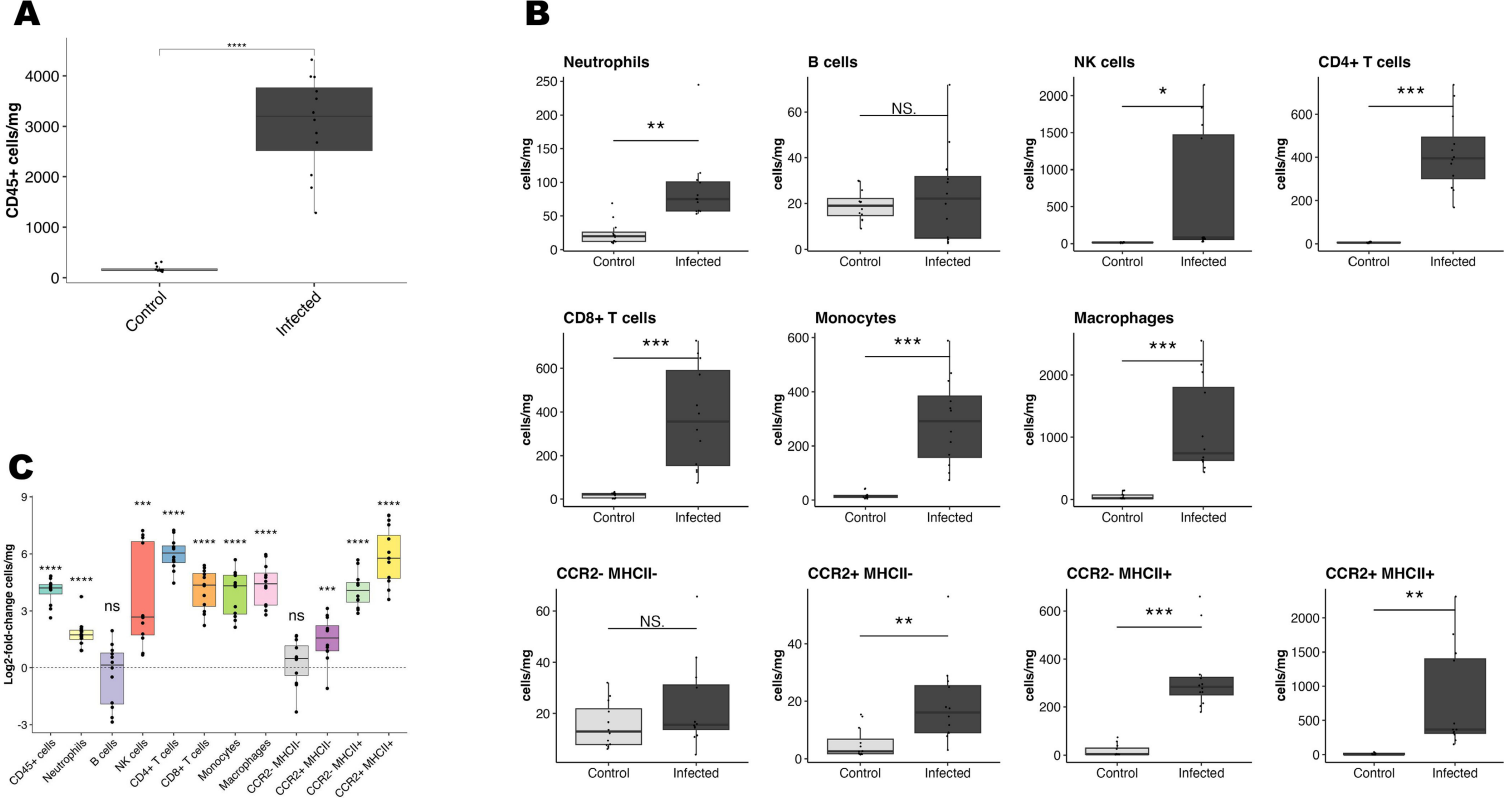
The intracardiac immune cell population during *T. brucei* infection is characteristic of immune-mediated myocarditis. (**A)** Flow cytometric quantification of CD45+ cells per milligram. At 28 dpi, CD45+ cells are markedly increased in the hearts of infected mice (*n* = 12) compared to uninfected age-matched controls, supportive of myocarditis (two-sided Student’s *t*-test, *P* = 4.6e-7). (**B)** Flow cytometric quantification of myeloid and lymphoid cell populations. The following cell types were quantified: neutrophils (CD45+, CD11b+, Ly6G+), B cells (CD45+, CD19+), NK cells (CD45+, NK1.1+), CD4+ T cells (CD45+, CD11b–, CD4+), CD8+ T cells (C45+, CD11b–, CD8+), monocytes (CD45+, Cd11b+, Ly6C high, CD64 low), total macrophages (CD45+, Cd11b+, Ly6C low, CD64 high), in addition to four macrophage subsets defined by the expression of CCR2 and MHCII. Most immune cell populations are significantly increased in the hearts of infected mice at 28 dpi. Statistical comparisons were made using one-way analysis of variance (ANOVA). *P < 0.05, **P < 0.01, ***P < 0.001. (**C)** Visualization of magnitude of changes in immune cell populations using the log2-fold change in cells per milligram compared to uninfected age-matched control mice calculated based on the average cells per milligram in the hearts of uninfected control mice.

We had identified myocarditis as a consequence of *T. brucei* infection, and previous research has indicated that the host immune response may contribute to *T. brucei-*associated cardiac pathology (29). To better understand the intracardiac immune response to *T. brucei*, we performed flow cytometry at 28 dpi to characterize the immune cell population in the heart. We measured total numbers of cells expressing CD45, a marker common to all nucleated hematopoietic cells, which is commonly used to assess the magnitude of inflammatory responses (42). Numbers of CD45+ cells per milligram of tissue were markedly elevated in the hearts of all infected mice at 28 dpi, supporting our histopathological finding of myocarditis (Fig. 5A).

Examining individual cell populations, most immune cell lineages were more abundant in *T. brucei*-infected hearts than in age-matched controls (Fig. 5B and C). In agreement with the functional data, most immune cell populations were slightly more elevated in males than in females, although the difference was not statistically significant for any cell lineage (Fig. S3G and H).

Numbers of both CD4+ T cells and macrophages were significantly increased in infected hearts, which is unsurprising, as these cell types are necessary for an effective immune response to African trypanosomes (Fig. 5B and C) (43). Of the different macrophage subsets, the CCR2^hi^MHC-II^hi^ population experienced the largest increase in numbers. This subset represents pro-inflammatory, monocyte-derived macrophages, indicating that *T. brucei* infection causes an influx of pro-inflammatory macrophages into the heart (44, 45). There was also a significant elevation of both CD8+ T cells and natural killer (NK) cells, even though cell-mediated immunity is relatively ineffective at responding to *T. brucei,* which is an extracellular parasite (44, 45).

## DISCUSSION

*T. brucei*-associated cardiac disease is an underappreciated and understudied aspect of African trypanosomiasis. Here, we report a reproducible murine model of *T. brucei* infection optimized to investigate acute and chronic *T. brucei*-associated cardiac disease. We demonstrate that this model recapitulates key aspects of *T. brucei*-associated cardiac disease in humans and natural hosts, including the presence of intracardiac parasites, myocarditis, EKG changes, and elevated NT-proBNP. Using this model, we learned that *T. brucei* infection results in parasite colonization of the myocardial extracellular space and elevation of NT-proBNP (Fig. 1 to 3). NT-proBNP elevation was initially present in the context of a preserved ejection fraction, but the infection eventually resulted in severe cardiac dysfunction as mice approached terminal endpoint (Fig. 1 and 2). At a histological level, we found that myocardial colonization by the parasite triggered a severe myocarditis characterized by cardiomyocyte damage (Fig. 4) and a multilineage inflammatory infiltrate (Fig. 5). These findings advance our understanding of the host–pathogen interactions in the heart, suggesting that cardiac dysfunction associated with *T. brucei* infection is an immune-mediated event, potentially providing valuable information to inform the development of novel targeted therapies for animals and humans affected by African trypanosomiases.

Animal models have been used to study *T. brucei* infection since the early 1900s, and the first attempts to use a murine model to investigate *T. brucei*-associated cardiac disease were published in the late 1970s (15, 29, 30). In these historical murine studies, investigators demonstrated cardiac lesions in infected mice and showed that immunosuppression was associated with a reduction in the size and frequency of inflammatory cardiac lesions (29). However, despite their seminal nature, these studies were limited in the scope of their investigation, primarily focusing on histopathology and lacking more detailed functional or immunological assessments. Moreover, the studies were performed using infection protocols and parasite lines that were not standardized, thus making their methods challenging to replicate. Our present work expands significantly on those historical studies as it presents a reproducible method to generate cardiac damage and dysfunction in a murine model, demonstrably recapitulating multiple aspects of natural disease.

We have found that mice infected with *T. brucei* initially exhibit elevation in NT-proBNP and electrocardiographic abnormalities in the context of a preserved left ventricular ejection fraction. At 28 dpi, when we initially assessed BNP, the mice had clear histological evidence of myocarditis. NT-proBNP is elevated in response to an increase in intracardiac filling pressures (35). In the context of a normal ejection fraction and cardiac pathology, BNP elevation is suggestive of heart failure with preserved ejection fraction (HFpEF), a clinical condition characterized by reduced cardiac performance and symptoms of heart failure despite a seemingly normal cardiac contractility (46). However, BNP elevation in the context of a normal ejection fraction has also been described in patients with acute myocarditis, likely as a result of stiffening of cardiac walls triggered by the intracardiac immune response (47, 48). The potential overlap between myocarditis with preserved ejection fraction and elevated BNP and HFpEF is an area that has not been properly investigated so far in the clinical setting. However, taken together, our findings might suggest that colonization of the cardiac interstitium by *T. brucei* might result in a stiffening of cardiac walls and elevation of cardiac pressures compatible with HFpEF, and that the myocarditic immune response triggered by the parasites likely plays a key role in this process. We hypothesize that as mice approach terminal endpoint, the cardiac pathology becomes severe enough to cause a decrease in ejection fraction. However, more definite conclusions require more detailed investigation into the functional impact of *T. brucei* infection at multiple timepoints.

At 28 dpi, electrocardiographic abnormalities included marked bradycardia, increased QT interval, grouped beating, and dropped beats. These findings are in agreement with reports from HAT patients, in which increased QT interval is the most common finding (16, 17). AV blocks have also been reported in a subset of HAT patients, while autopsies have found inflammation of the sinoatrial node, suggesting a disruption of the cardiac conduction system (49). Interestingly, we did not observe premature ventricular contractions (PVCs), which have been observed in a rat model of African trypanosomiasis (26). However, the relatively short recording period in our study (1 minute per animal) may have hampered measurements of PVCs compared to the rat model, in which 30-minute EKG recordings were analyzed (26).

We used flow cytometry to investigate the intracardiac immune milieu during *T. brucei* infection. Previous studies in humans and large animal models have found that the major immune effectors during *T. brucei* infection are B cells, CD4+ T cells, and macrophages, which are all necessary for a protective anti-trypanosomal immune response (43). On the other hand, CD8+ T cells and NK cells are thought to be mediators of pathological inflammation in *T. brucei* infection, and their activation is associated with worse clinical outcomes (43, 50, 51). In agreement with our histopathological finding of myocarditis, we found that most intracardiac immune cell populations were elevated, especially CD4+ and CD8+ T cells, macrophages, and NK cells. The most highly elevated macrophage subset was the CCR2^hi^MHC-II^hi^ population, which represents pro-inflammatory, monocyte-derived macrophages (44, 45). Such pro-inflammatory macrophages are necessary for an effective immune response to *T. brucei* (43). However, increased activation of anti-inflammatory, alternatively activated macrophages during chronic *T. brucei* infection is important for long-term survival in experimentally infected animals, due to their ability to attenuate pathological inflammation (45, 46). The large numbers of intracardiac CCR2^hi^MHC-II^hi^ in our model suggest that an imbalance between pro-inflammatory and anti-inflammatory macrophages could be a contributor to myocarditis in our model. The elevation of CD8+ T cells and NK cells in the heart is further suggestive of immunopathology as a contributor to cardiac damage. Increases in these cell populations are suggestive of a response to intracellular antigens, although *T. brucei* is entirely extracellular throughout its life cycle (50). Because CD8+ T cells are not a major feature of the immune response to extracellular pathogens, we hypothesize that their elevation in our model indicates a response to cardiac self-antigens, indicating an auto-immune component of the observed myocarditis. This hypothesis is corroborated by other evidence suggesting that *T. brucei*-associated organ damage has an auto-immune component. A recent study found that meningeal B cells produce high-affinity autoantibodies during *T. brucei* infection, suggesting that autoimmune responses may be a component of *T. brucei*-associated pathology in multiple tissues (52). Clinically, cardiomyopathy patients in Cameroon have been found to have high levels of both anti-trypanosomal and anti-heart antibodies, further indicating a connection between *T. brucei* infection and autoimmune heart disease (18).

Furthermore, we hypothesize that immunopathology in the heart during *T. brucei* infection follows a similar process to the pathogenesis of Chagas cardiomyopathy, caused by the New World trypanosome *T. cruzi*. The leading theory to explain the pathogenesis of Chagas disease posits that parasite colonization of the heart induces inflammation and release of self-antigens, resulting in a breakdown of self-tolerance and immune destruction of cardiomyocytes (53). With chronicity, loss of cardiac muscle and replacement with scar tissue cause a collapse in cardiac function, ultimately ending in the characteristic Chagas cardiomyopathy (53). Our findings suggest that similar mechanisms might be at play during *T. brucei* infection.

There was no increase in the B cell population in the heart. This is somewhat surprising, because antibodies are the major mediators of immune clearance of *T. brucei*, and B cells are crucial for systemic anti-trypanosomal immunity (43). However, our findings are consistent with other tissue-specific flow cytometry experiments during *T. brucei* infection, which have identified no changes in the number of B cells in the lungs or adipose bodies of *T. brucei*-infected mice (14, 54). In contrast, other models of myocarditis and heart failure show that intracardiac B cell populations are increased and play a major pathologic role during viral myocarditis and myocardial infarction (55–60). It is possible that B cell activation plays a role in *T. brucei*-associated cardiac disease, even if there is no expansion of local populations.

We found limited evidence of a sex-specific difference in *T. brucei*-associated cardiac disease. Male mice exhibited higher NT-proBNP at 33 dpi, higher numbers of intracardiac immune cells, and more severe echocardiographic and EKG changes than females, although these differences were not statistically significant. Our findings are in agreement with evidence that HAT is more severe in men than in women, as well as recent findings that indicate that *T. brucei* causes more severe weight loss and adipose wasting in male rodents (61, 62). Our findings are also consistent with data indicating that myocarditis is more common in men than in women (63, 64). However, interpretation was hampered by the relatively small differences in parameters between male and female mice, which would require larger group sizes to adequately explore. Further work will be needed to confirm and expand on these observations and dissect the mechanistic basis of sex differences in HAT.

Although our work provides convincing evidence of *T. brucei* as a cardiac parasite, there are important limitations to be noted. In particular, there are specific limitations to the mouse model we used. We chose the C57Bl/6J strain due to its established use as a *T. brucei* model and its lack of background cardiac lesions compared to other mouse strains, especially the BALB/c (31, 65). However, it will be necessary in the future to confirm that these findings are consistent with other mouse strains widely used by parasitologists. In addition, the short time-course of *T. brucei* infection in mice—weeks to months, compared to months to years in humans—makes it challenging to evaluate long-term changes. *T. b. gambiense* can be used to model long-term infection in mice, although the higher biosafety requirements of working with human-infective trypanosomes make this strain impractical for many researchers (34). Our study was also affected by relatively small sample sizes. Further work will be necessary to characterize cardiac disease over a longer time-frame and with larger samples. Similarly, we initiated our infections using a small, clonal inoculum of bloodstream form parasites injected intravenously. This is in contrast with the thousands of metacyclic form parasites that are injected intradermally by tsetse bite during a natural infection. It will be important to determine whether the route of infection or the inoculum size has any effect on cardiac pathology.

Much about *T. brucei*-associated cardiac disease remains unknown, and there are serious gaps in our current knowledge, which this animal model could be used to investigate. Our findings raise concerns about long-term, post-treatment consequences of *T. brucei* infection. Most HAT patients are identified and treated, permanently clearing infection. However, the severity of the myocarditis reported by ourselves and others suggests that heart dysfunction might be only loosely connected with parasite presence. (20, 22–25, 29, 39, 40). If so, anti-parasitic treatment would not fully alleviate the cardiac symptoms of HAT, because self-directed inflammation could continue even with the removal of the inciting stimulus. Moreover, this suggests that immunomodulatory therapies could be considered for treatment of both humans and cattle to suppress inflammation that could contribute to persistent heart disease. There is a necessity to investigate the long-term and post-treatment consequences of *T. brucei*-associated myocarditis to determine whether HAT patients are at an increased lifetime risk of heart failure. In addition, the effect of *T. brucei* subspecies on cardiac disease remains to be investigated. Our mouse model most closely recapitulates HAT due to *T. b. rhodesiense* infection. However, currently, the majority of human cases are caused by *T. b. gambiense*, which often causes a disease of a more chronic character than *T. b. rhodesiense* (2). Although there is clinical evidence that all *T. brucei* subspecies cause cardiac disease, the differences in cardiac manifestations between subspecies are uncertain (16, 21, 66).

In summary, we describe the first reproducible, optimized, clinically relevant murine model of *T*. *brucei*-associated cardiac disease, a feature of African trypanosomiasis that is often overlooked in current textbooks and clinical resources. In the context of this model’s characterization, we produced evidence suggesting that *T. brucei*-associated cardiac damage is an immune-mediated event. Future studies with this model will be instrumental in identifying potential treatments for cardiac manifestations of African trypanosomiasis, a disease that continues to take an extensive human and economic toll in large regions of the world. In particular, the potential for immune-mediated disease indicates that this model could be used to investigate the use of immunomodulatory therapies to alleviate cardiac disease during African trypanosomiasis. Corroboration of our findings by further studies would indicate the potential use for current immunomodulatory drugs as adjunct therapies for African trypanosomiasis, providing a tool to improve health outcomes.

## MATERIALS AND METHODS

### Sex as a biological variable

For all experiments, data from both male and female mice were collected and analyzed.

### Mouse and parasite strains and infection

Male and female C57Bl/6J mice (WT, strain# 000664 Jackson Laboratory) between 7 and 10 weeks old were each infected by intravenous tail vein injection with ∼5 pleiomorphic bloodstream form EATRO 1125 AnTat1.1E 90-13 *T. brucei* parasites (67). The parasite stocks used were maintained as blood stabilates in HMI-9 plus 10% glycerol. Stabilates were thawed and immediately diluted in HMI-9 media prior to injection. To quantify parasitemia, blood was collected from the tail vein every 2 days starting at 4 dpi, and parasites were counted via hemocytometer. Blood (125 μL) was collected by a submandibular bleed at terminal endpoint (28 or 33 dpi). Unless otherwise described, mice were euthanized at terminal endpoint by intraperitoneal injection of ketamine/xylazine and were perfused with 50 mL of phosphate-buffered saline (PBS)-Glucose (0.055 M D-glucose) with heparin to remove intravascular parasites. Mice were euthanized when they reached 10^9^ parasites per milliliter of blood, lost >20% of body weight, or were assessed to be clinically terminal.

For immunofluorescence experiments, mice were infected intravenously with ~5 AnTat1.1E chimeric triple reporter *T. brucei* parasites, which express tdTomato (36). Parasitemia was measured as previously described. At 14 dpi, mice were sacrificed as previously described.

For flow cytometry experiments, mice were infected, and parasitemia was measured as previously described. Mice were sacrificed at 28 dpi using CO_2_ euthanasia.

### Echocardiography

At 28 dpi, echocardiography was performed on sedated mice by a blinded investigator using a Vevo 2100 Ultrasound System (VisualSonics Inc.) as described previously (68).

At 33 dpi, echocardiography was performed on awake mice using a 40-MHz transducer (VisualSonics Inc.) as described previously (69).

### Electrocardiography

At 28 dpi, EKG was performed on unanesthetized mice as previously described (70). The entire procedure was performed in approximately 1 minute per mouse using a PowerLab data acquisition system (model ML866, ADInstruments, Colorado Springs, CO, USA) and Animal Bio Amp (model ML136, ADInstruments). EKG data were analyzed using LabChart software (ADInstruments). The lead II tracings were used for analysis.

### Heart digestion

Heart digestion was performed using a technique previously described (71). Briefly, after sacrifice, mice were perfused with 3 mL Hank’s Balanced Salt Solution (HBSS). Heart tissue (60 mg) was placed in a 15-mL tube with 3 mL of HBSS. Digestion was performed for half an hour with 300 units of DNase I (Sigma-Aldrich), 625 units of collagenase II (Sigma-Aldrich), and 50 units of hyaluronidase II (Sigma-Aldrich). After digestion, the tubes were centrifuged at 250 × *g* for 5 minutes at 4°C, the supernatant was removed, and the pellet was resuspended in 5 mL ACK lysis buffer (Invitrogen) and incubated for 5 minutes at room temperature to remove red blood cells. Suspended cells were passed through a 50-µm filter and then prepared for flow cytometry.

### Flow cytometry

Flow cytometry was performed using a technique previously described (71). Cell suspensions were labeled with fluorescently conjugated antibodies (Table S1). Flow cytometry was performed on an Aurora Spectral Flow Cytometer (Cytek), and analyses were performed using FlowJo version 10.8.1 (Becton Dickinson).

Immune cells were defined as CD45+. From CD45+ cells, CD11b+ and CD11b–/CD11b+ cells were gated. From this gate, Ly6G+/CD11b+ cells were gated and identified as neutrophils. From CD11b+/Ly6G– cells, monocytes and macrophages were identified, respectively, as Ly6C high, CD64 low and CD64 high, Ly6C low. Macrophages were further gated according to the expression of MHC-II and CCR2 in four quadrants/populations. The CD11b– population was then sub-gated into CD4+ T cells, CD8+ T cells. CD19+ B cells and Nk 1.1+ NK cells were identified from the CD45+ population. An example gating strategy is presented in Fig. S5.

### NT-proBNP ELISA

At 28 and 33 dpi, plasma was collected from mice via cheek-bleed. NT-proBNP plasma levels were measured using the LS-F34395 Mouse NT-proBNP Sandwich ELISA Kit (LSBio) according to the manufacturer’s instructions.

### Histopathology

For histopathology experiments, animals were infected and sacrificed at 28 dpi as previously described. Hearts were placed in 10% neutral-buffered formalin and were fixed at room temperature for 48 hours. The heart was cut in half longitudinally and sectioned by the Johns Hopkins Oncology Tissue and Imaging Services Core. To evaluate inflammation, hearts were stained with hematoxylin and eosin, and imaged with 4×, 10×, and 40× objectives using a Nikon Eclipse E400 light microscope (Nikon). Cardiac lesions were graded by a blinded veterinary pathologist using both the previously established Dallas Criteria and a semiquantitative scoring system for myocarditis in rodents (Table 2) (37, 38). Briefly, the severity and extent of lesions were graded using a five-point system from 0 to 4. A zero score indicates no infiltration. A “1” score indicates very limited focal distribution of myocardial lesions. A “2” score indicates multiple lesions. A “3” score indicates multiple regions with confluence and extensive necrosis. A “4” score indicates lesions affecting most of the observed tissue. Images were captured using an Excelis 4K camera (Unitron) and were processed using ImageJ v1.53 image analysis software (NIH). All methods followed published guidelines for experimental pathology (72). To evaluate cardiac fibrosis, additional longitudinal sections of hearts were stained with Masson’s Trichrome and scanned at 200× magnification using an Axios Z.1 scanner (Zeiss). The percentage of collagen in each heart section was quantified using the AFAT (41).

### Immunofluorescence

For immunofluorescence experiments, 8-week-old female C57Bl/6J mice were infected intravenously with five triple marker *T. brucei* parasites (36). At 14 dpi, mice were euthanized and perfused as previously described. Heart was collected and fixed in 4% paraformaldehyde in PBS for 12 hours at 4°C. Post-fixation, tissues were sectioned longitudinally, frozen embedded in O.C.T. Compound (Tissue-Tek), and cut using cryostat microtome into 10-µm sections by the Johns Hopkins Oncology Tissue and Imaging Services Core.

The following antibodies were applied to the sections: rat anti-mouse CD-31 (Santa Cruz Biotechnology) followed by goat anti-rat Alexa Fluor 488 (Cell Signaling Technology). Coverslips were mounted using ProLong Gold (Life Technologies). Tissues were imaged with 4×, 10×, and 20× objectives using a Nikon Eclipse 90i fluorescence microscope (Nikon) and X-Cite 120 fluorescent lamp (Excelitas) with an ORCA-ER digital CCD camera (Hammamatsu) and ImageJ v1.53 image analysis software (NIH). Image collection and analysis followed published guidelines for rigor and reproducibility in immunofluorescence (73).

### Statistics

Unless otherwise described, figures were generated, and statistical analyses were performed using the Tidyverse package in R version 4.2.3 (74).

## Data Availability

Values for all data points in graphs are reported in the Supporting Data Values file.

## SUPPLEMENTAL MATERIAL

Supplemental figures and tables spectrum.01623-24-s0001.pdf Figure S1 to S5; Table S1.

Supporting data values spectrum.01623-24-s0002.xlsx Raw data used for figures.

ASM does not own the copyrights to Supplemental Material that may be linked to, or accessed through, an article. The authors have granted ASM a non-exclusive, world-wide license to publish the Supplemental Material files. Please contact the corresponding author directly for reuse.
