# Supplementary material for: A murine model of Trypanosoma brucei-induced myocarditis and cardiac dysfunction
Source: Microbiol Spectr. 2025 Jan 10;13(2):e01623-24. doi: 10.1128/spectrum.01623-24 (PMC11792545; doi:10.1128/spectrum.01623-24)
Supplement: Supplemental figures and tables — Figure S1 to S5; Table S1. [file spectrum.01623-24-s0001.pdf]

Supplemental Materials

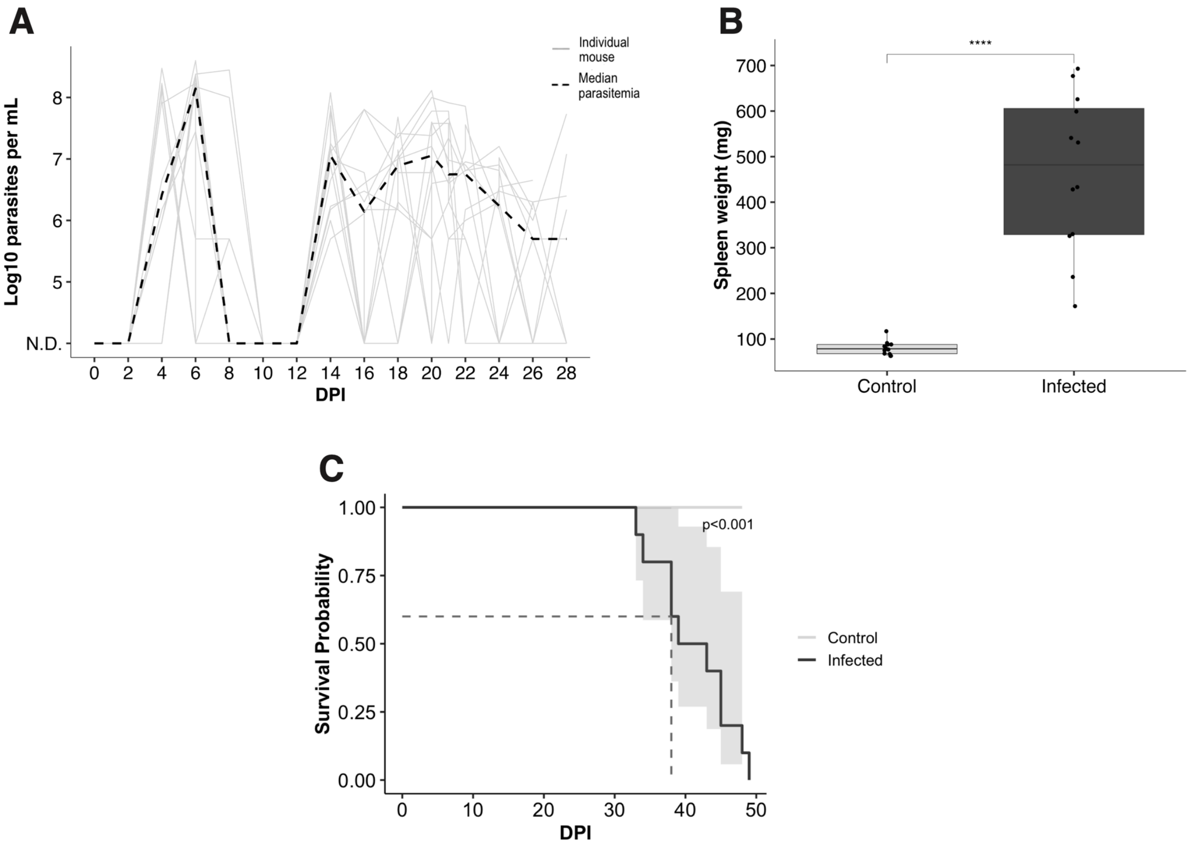

Supplemental Figure 1. *T. brucei* infection causes waxing and waning waves of parasitemia and massive splenomegaly, with terminal endpoint reached within 50 dpi

A. Quantification of median and individual parasitemia in a representative experiment. Parasitemia first becomes detectable at 4-6 dpi.

B. Weight of spleens of infected mice at 28 dpi(n=6) compared to uninfected, age-matched controls. Infected mice exhibit significant splenomegaly (two-sided Student's t-test, p=3.7E-06).

C. Kaplan-Meier survival curve of mice infected with *T. brucei* vs. uninfected control animals. Infected mice have a median survival time=39 dpi (log-rank test, p=4.32E-05), indicated by the dotted line.

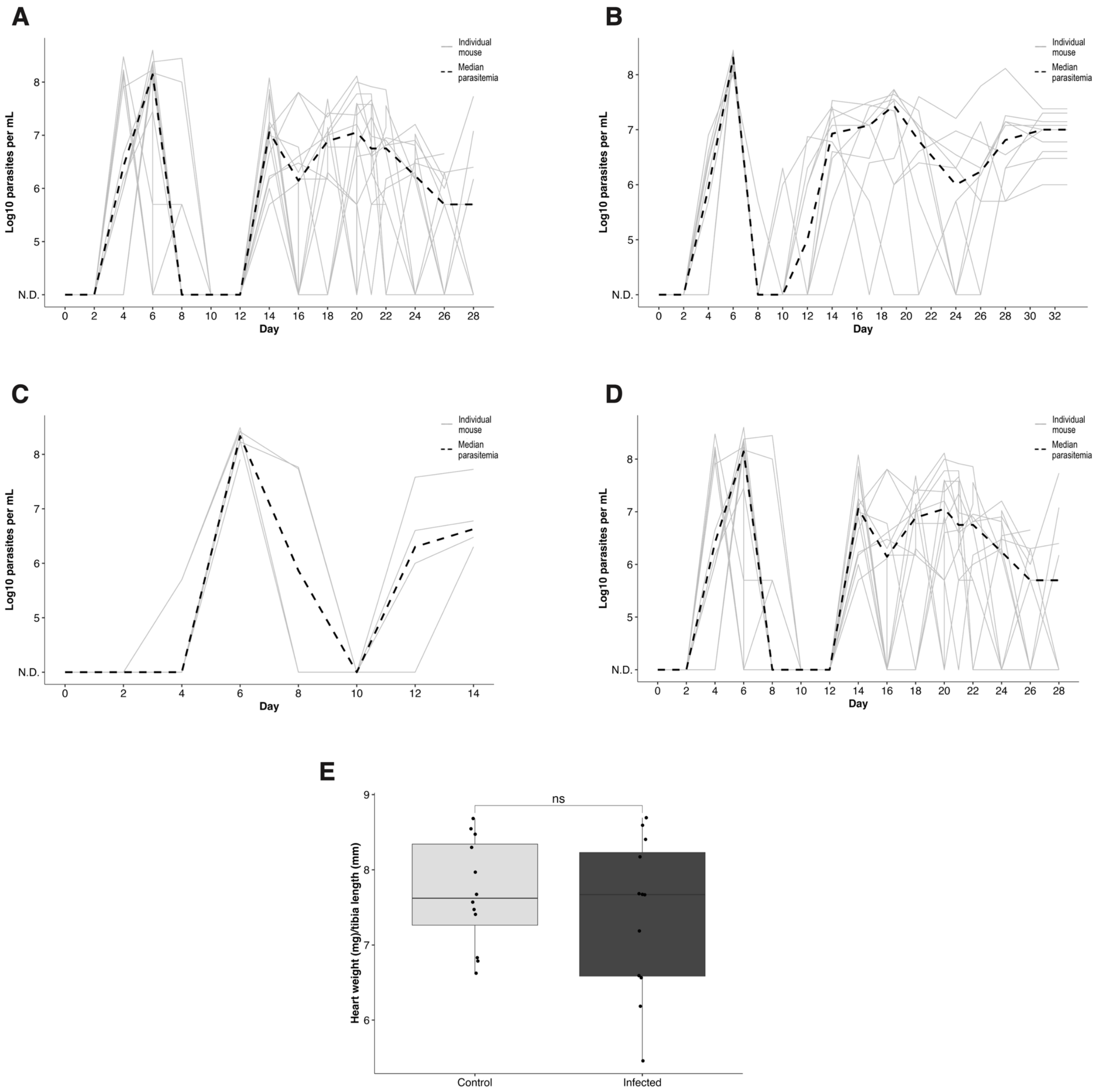

Supplemental Figure 2: Quantification of median and individual parasitemia for all experiments and heart weight at 28 dpi

A. Parasitemia of mice used for data collection at 28 dpi (NT-proBNP, echocardiography, histopathology)
B. Parasitemia of mice used for data collection at 33 dpi (NT-proBNP, echocardiography)
C. Parasitemia of mice used for data collection at 6 and 14 dpi (Immunofluorescence)
D. Parasitemia of mice used for survival curve and 28 dpi ECG
E. Heart weight in mg normalized to tibia length in mm of mice sacrificed at 28 dpi

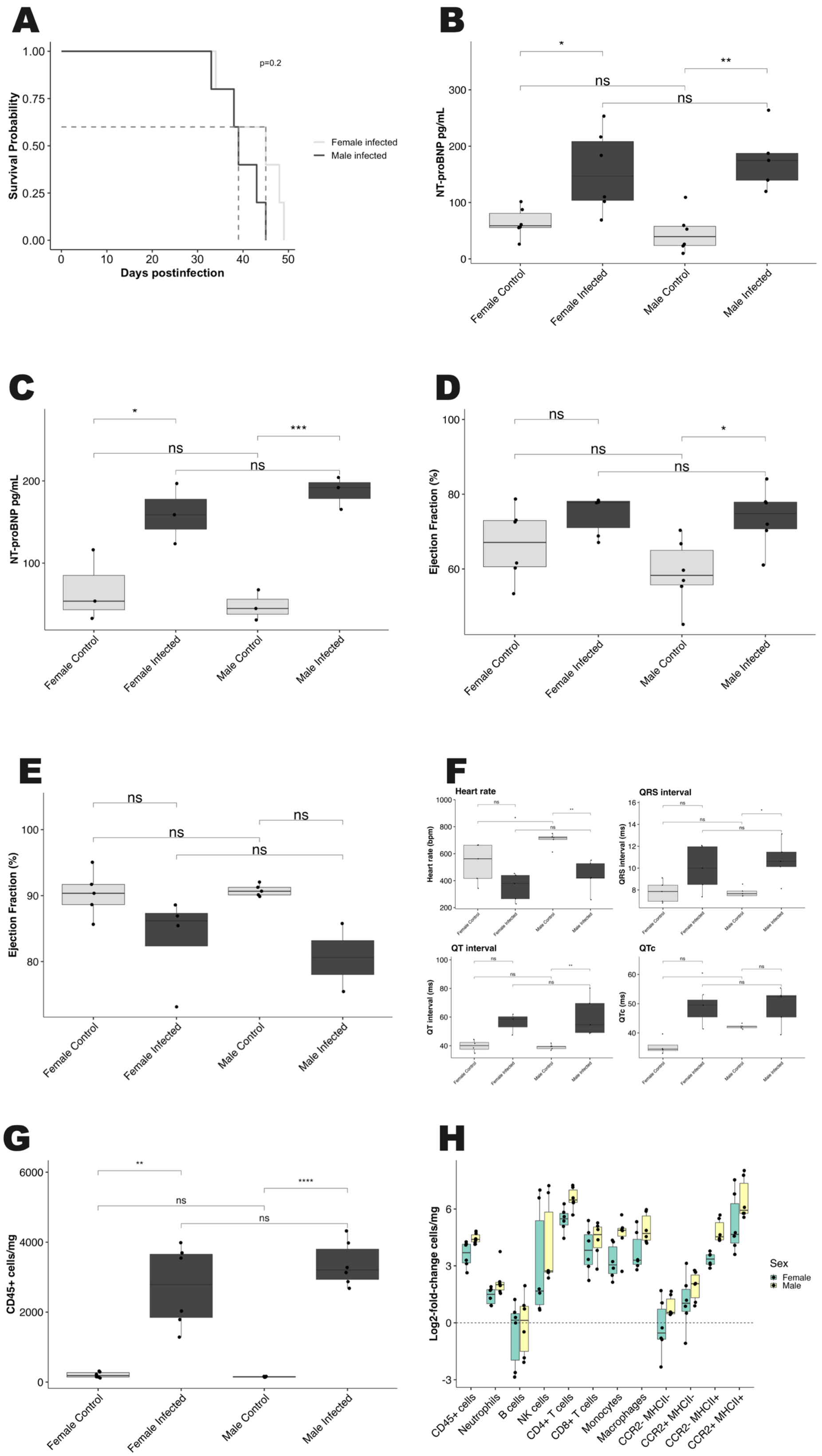
A
Survival Probability
Days postinfection
p=0.2
Female infected
Male infected
B
NT-proBNP pg/mL
Female Control
Female Infected
Male Control
Male Infected
C
NT-proBNP pg/mL
D
Ejection Fraction (%)
E
Ejection Fraction (%)
F
Heart rate
QRS interval
QT interval
QTc
G
CD45+ cells/mg
H
Log2-fold-change cells/mg
CD45+ cells
Neutrophils
B cells
NK cells
CD4+ T cells
CD8+ T cells
Monocytes
Macrophages
CCR2- MHCII-
CCR2+ MHCII-
CCR2- MHCII+
CCR2+ MHCII+
Sex
Female
Male

Supplemental Figure 3: Experimental parameters of cardiac function and immunity, separated by sex

A. Survival curve separated by sex. Median survival time is 39 dpi for males and 45 dpi for females, p=0.2.
B. Plasma NT-proBNP measured at 28 dpi, compared between males and females (n=6 for each group)
C. Plasma NT-proBNP measured at 33 dpi (n=3 for each group)
D. Ejection fraction measured via sedated echocardiography at 28 dpi (n=6 for each group)
E. Ejection fraction measured via awake echocardiography at 33 dpi (n=4 for each group except Male Infected, for which n=2)
F. Electrocardiographic changes divided by sex (n=5 for each group)
G. Intracardiac CD45+ cells/mg measured via flow cytometry at 28 dpi (n=6 for each group
H. Log2-fold changes of all immune cell populations in infected mice at 28 dpi (n=6 for each group)

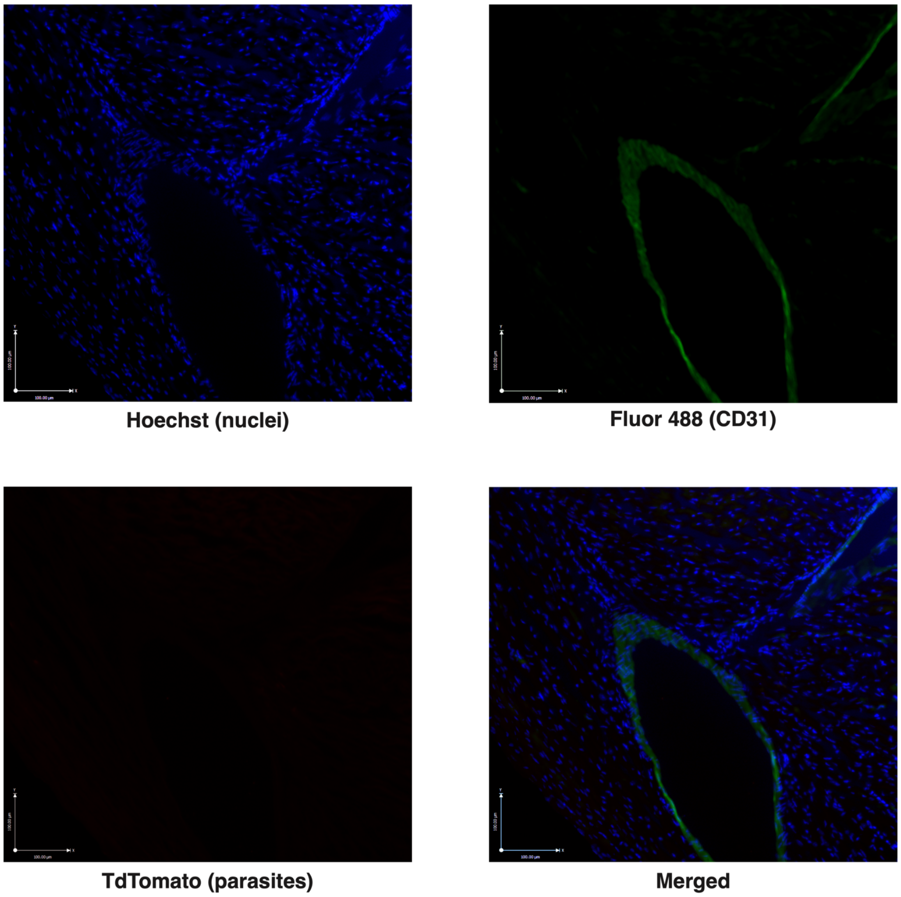

Supplemental Figure 4: Representative immunofluorescence microphotograph of the cardiac ventricle of an uninfected mouse at 200x magnification.

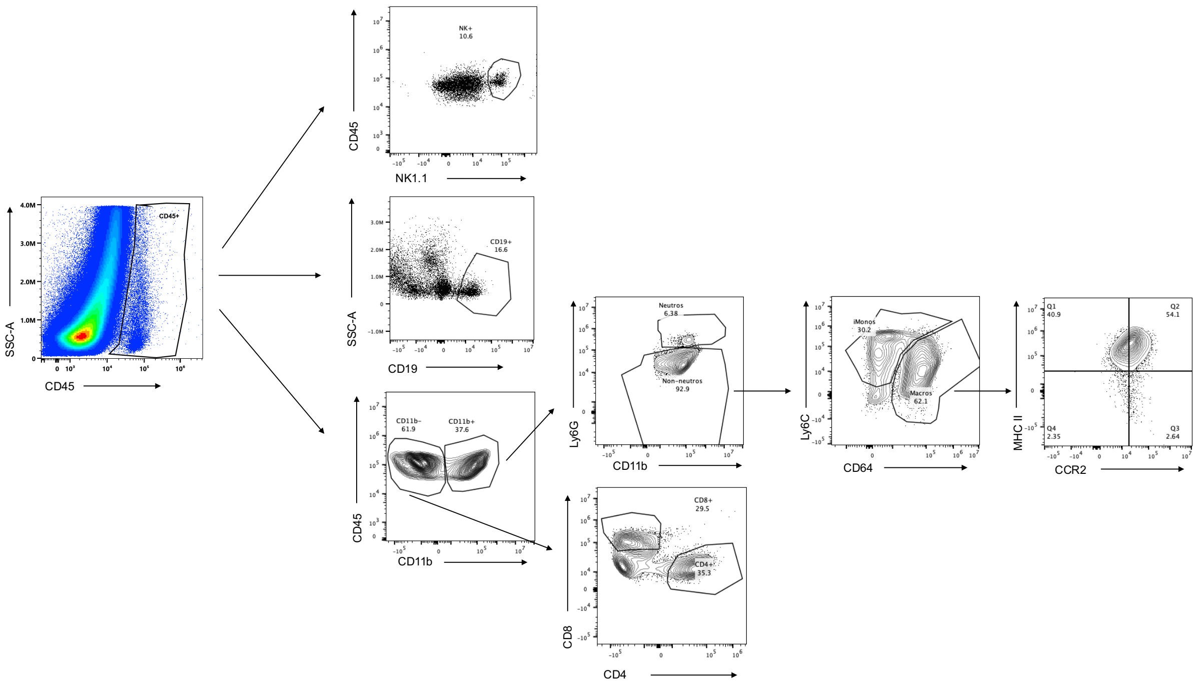

Supplemental Figure 5: Flow cytometry gating strategy.

Supplemental Table 1: Flow cytometry antibodies

Antibody	Brand	Fluorophore	Clone	Cat. No.
CD45	Biolegend	PerCP/Cy5.5	30-F11	103132
CD19	Biolegend	BV421	6D5	115537
CD11b	Biolegend	AF700	M1/70	101222
CD4	Biolegend	SparkNIR 685	GK1.5	100475
CD8a	Biolegend	BV785	53-6.7	100749
Ly6C	Biolegend	BV650	HK1.4	128049
Ly6G	Biolegend	APC Cy7	1A8	127623
CD64	Biolegend	PECy7	X54-5/7.1	139313
CCR2	Biolegend	APC	QA18A56	160103
MHCII	Biolegend	BV711	M5/114.15.2	107643
